# Temporal trends and risk factors for extended-spectrum beta-lactamase-producing *Escherichia coli* in adults with catheter-associated urinary tract infections

**DOI:** 10.1186/s13756-014-0039-y

**Published:** 2014-12-27

**Authors:** Joseph T Spadafino, Bevin Cohen, Jianfang Liu, Elaine Larson

**Affiliations:** Department of Epidemiology, Columbia University Mailman School of Public Health, 722 West 168th Street, New York, NY 10032 USA; Columbia University School of Nursing, 630 West 168th Street, New York, NY 10032 USA

**Keywords:** Catheter-associated urinary tract infections, Extended-spectrum beta-lactamase-producing *Escherichia coli*, Antimicrobial resistance

## Abstract

**Background:**

Extended-spectrum beta-lactamase (ESBL)-producing *Escherichia coli* cause up to 10% of catheter-associated urinary tract infections (CAUTI). We report changes in ESBL prevalence among CAUTIs in an adult acute care hospital from 2006-2012 and describe factors associated ESBL-production among *E. coli* CAUTI.

**Findings:**

Data on patients ≥18 years discharged from a 647-bed tertiary/quaternary care hospital (2006-2012), a 221-bed community hospital (2007-2012), and a 914-bed tertiary/quaternary care hospital (2008) were obtained retrospectively from an electronic database (N = 415,430 discharges). Infections were identified using a previously validated electronic algorithm. Information on medical conditions and treatments were collected from electronic health records and discharge billing codes. A case-control design was used to determine factors associated with having a CAUTI caused by an ESBL-producing *E. coli* versus a non-ESBL-producing *E. coli*. Changes in yearly proportion of ESBL *E. coli* CAUTI at the 647-bed tertiary/quaternary care hospital were evaluated. ESBL increased from 4% in 2006 to 14% in 2012, peaking at 18% in 2009. Prior antibiotic treatment and urinary tract disease significantly increased odds of ESBL.

**Conclusions:**

This study provides evidence that treatment with beta-lactam and non-beta-lactam antibiotics is a risk factor for acquiring ESBL-producing *E. coli* CAUTI, and the prevalence of this organism may be increasing in acute care hospitals.

## Introduction

*Escherichia coli* is the most common causative agent of catheter-associated urinary tract infections (CAUTI; >20%) [1]. Extended-spectrum beta-lactamase (ESBL)-producing strains of *E. coli*, while representing a small percentage (<10%), are particularly concerning because they confer resistance to a myriad of antibiotics including penicillins and third generation cephalosporins, and because their prevalence has been increasing in community and hospital settings during recent years [2,3]. The purpose of this study is to describe changes in prevalence and factors associated with CAUTI caused by ESBL-producing *E. coli* in three adult acute care hospitals from 2006 through 2012.

## Methods

Data on all patients ≥18 years discharged from three New York City hospitals within a single network were obtained retrospectively from a larger electronic database as part of an NIH-funded study (Distribution of the Costs of Antimicrobial Resistant Infections, NR010822). The study was approved by the Institutional Review Board of Columbia University Medical Center. Data were available from 2006-2012 for a 647-bed tertiary/quaternary care hospital, from 2007-2012 for a 221-bed community hospital, and from 2008 for a 914-bed tertiary/quaternary care hospital (N = 415,430 discharges). As described in detail in Apte et al. [4], the database contained information from a number of electronic sources, including patients’ electronic health, discharge, laboratory, and medication administration records. The discharge data provided admission and discharge dates, International Classification of Diseases Ninth Revision Clinical Modification (ICD-9-CM) codes for primary and secondary diagnoses present on admission, age, gender, comorbidities, and surgical procedures. The clinical data sources provided time stamped information on catheterization and medications.

CAUTI were defined based on National Healthcare Safety Network (NHSN, http://www.cdc.gov/nhsn/about.html) guidelines (positive urine culture >48 hours after urinary catheterization) and identified using previously validated computerized algorithms [4,5]. If a patient had multiple CAUTIs during the study period, only the first was included. We performed a case-control study to determine factors associated with having a CAUTI caused by an ESBL-producing *E. coli* versus a non-ESBL-producing *E. coli*. First, we determined the bivariable associations between ESBL production and age, sex, comorbidities including diabetes mellitus, HIV, urinary tract disease, and malignancies, Charlson Comorbidity Index score (http://www.uroweb.org/fileadmin/livesurgery/Charlson_Comorbidity_Index.pdf), urinary tract procedures, days of catheterization, and antibiotic treatment received during the patient’s hospitalization prior to infection onset. All variables significantly associated (p ≤ 0.05) with ESBL production were included in a multivariable logistic regression model. We evaluated changes in yearly proportion of ESBL *E. coli* CAUTI at the 647-bed tertiary/quaternary care hospital, for which data were available from 2006-2012, using the Cochran-Armitage test for trend. All statistical analyses were performed using SAS version 9.2 (SAS Institute Inc., Cary, NC).

## Results

**Figure 1:**
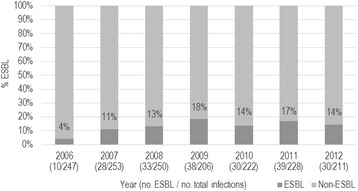
**Percentage by year of** ***Escherichia coli*** **CAUTI which were ESBL**-**positive in an adult tertiary**/**quaternary care hospital,** **2006**-**2012.** Yearly change in proportion is significant using Cochran-Armitage test for trend (p < 0.0001).

During the seven-year study period a total of 2,164 patients (1,616 women, 74.7%, and 548 men, 25.3%) developed a CAUTI with *E. coli* as the primary infecting pathogen, and 271 (12.5%) were ESBL-producing. The proportion of *E. coli* CAUTI that were ESBL-producers in the 647-bed tertiary/quaternary care hospital increased from 4% in 2006 to 14% in 2012, peaking at 18% in 2009 (p < 0.0001; Figure 1). Over half of patients with CAUTI were diagnosed within 4 days of catheter insertion, over three-quarters within 8 days, and over 98% (n = 2,123) within 30 days. Descriptive characteristics and antibiotics administered prior to CAUTI onset are summarized for cases and controls in Table 1.

**Table 1:** **Factors associated with catheter**-**associated urinary tract infections caused by extended**-**spectrum beta**-**lactamase** (**ESBL**)-**producing** ***Escherichia coli*** **versus non**-**ESBL**-**producing** ***E. coli***

**Variable**	**ESBL-** **producing** ***E. coli*** **isolates** **(N =** **271)**	**Non**-**ESBL**-**producing** ***E. coli*** **isolates** **(N =** **1893)**	**Total** **(N = ** **2164)**	**Bivariable analysis p-** **value** ^**a**^	**Multivariable analysis Odds Ratio** **(95% ** **CI)**
Mean (range) age, in years	70.4 (24-98)	67.5 (18-103)	67.9 (18-103)	*0.003^b^	1.01 (1.00, 1.02)
Gender (reference, female)				0.01	1.22 (0.91, 1.65)
Male (n (%))	86 (31.7%)	462 (24.4%)	548 (25.3%)		
Female (n (%))	185 (68.3%)	1431 (75.6%)	1616 (74.7%)		
HIV Positive	5 (1.9%)	12 (0.6%)	17 (0.8%)	0.052^c^	2.09 (0.66, 6.55)
Diabetes	89 (32.8%)	522 (27.6%)	611 (28.2%)	0.07	NA
Urinary Tract Disease	226 (83.4%)	1420 (75.0%)	1646 (76.1%)	0.003	1.43 (1.01, 2.03)
Malignancy	59 (21.8%)	363 (19.2%)	422 (19.5%)	0.31	NA
Mean (range) Duration of Catheterization Prior to CAUTI Onset, in days (Mean)	7.74 (0-78)	6.32 (0-124)	6.50 (0-124)	*0.03^b^	0.99 (0.98, 1.01)
Urinary Tract Procedure	26 (9.59%)	113 (5.97%)	139 (6.42%)	0.02	1.43 (0.88, 2.32)
Mean (range) Charlson Comorbidity Score	7.73 (0-35)	6.76 (0-51)	6.88 (0-51)	0.001^d^	1.03 (0.99, 1.06)
Antibiotics Received Prior to CAUTI Onset	174 (64.2%)	804 (42.5%)	978 (45.2%)	<0.0001	NA
Antibiotic received**					
Aminoglycoside	27 (9.9%)	73 (3.9%)	100 (4.6%)	<0.0001	5.42 (1.77, 16.55)
Cephalosporin, 1^st^ Gen.	66 (24.4%)	346 (18.3%)	412 (19.0%)	0.02	2.34 (1.63, 3.36)
Cephalosporin, 3^rd^ Gen.	23 (8.5%)	29 (1.5%)	52 (2.4%)	<0.0001	2.89 (0.79, 10.67)
Macrolide	22 (8.1%)	69 (3.7%)	91 (4.2%)	0.001	2.39 (1.12, 5.08)
Penicillin, 2^nd^ Gen.	17 (6.3%)	91 (4.8%)	108 (5.0%)	0.30	NA
Penicillin, 4^th^ Gen.	50 (18.5%)	173 (9.1%)	223 (10.3%)	<0.0001	2.65 (1.48, 4.76)
Vancomycin	59 (21.8%)	203 (10.7%)	262 (12.1%)	<0.0001	3.40 (2.26, 5.12)

*CAUTI*, catheter-associated urinary tract infection; *ESBL*, extended-spectrum beta-lactamase; *NA*, not included in multivariable model.

^a^Chi-Square test unless otherwise denoted; ^b^T-test; ^c^Fisher’s Exact Test; ^d^Wilcoxon-Mann-Whitney Test.

*F-Test for Equality of Variances indicated unequal variance and appropriate p-value was used.

** In the multivariable model, antibiotics were examined as a seven-level categorical variable. Reference category: No antibiotics prior to CAUTI onset.

Results of the case control study are presented in Table 1. In the crude analyses, cases were significantly older than controls (70.4 and 67.5 years, respectively) and had greater Charlson Comorbidity scores (7.06 and 6.44, respectively). Male sex, preexisting urinary tract disease, having a urinary tract procedure, and longer duration of catheterization prior to CAUTI onset were also associated with increased odds of ESBL production. Receiving any antibiotic prior to CAUTI onset was significantly associated with ESBL production, and six antibiotics specifically were administered significantly more often in cases than in controls: aminoglycosides, first-generation cephalosporins, third-generation cephalosporins, macrolides, fourth-generation penicillins, and vancomycin. In the multivariable model, age, urinary tract disease, and receipt of aminoglycosides, first-generation cephalosporins, macrolides, fourth-generation penicillins, and vancomycin were significantly associated with ESBL production.

## Discussion

Though several studies have examined risk factors for ESBL emergence in hospitalized and non-hospitalized patients with urinary tract infections (UTI), these studies were community-based and not focused on catheterized patients [2,3,6-8]. Since CAUTI may result in antibiotic susceptibility patterns different from those of non-catheter-related UTI, understanding the prevalence trends and risk factors for ESBL emergence unique to this population is important for the development and implementation of effective prevention efforts [9]. The prevalence of ESBL-producing strains in this sample (12.2%) was slightly higher than reported in previous studies which included both catheterized and non-catheterized patients (7-10.9%) [6,7,10], but the overall increase in ESBL prevalence over time is consistent with other reports [2,3,10]. An analysis of trends in gram-negative bacterial resistance among patients with UTI from a nationally representative sample of US hospitals between 2000 and 2009 found a threefold increase in cases of ESBL-producing *E. coli*, similar to our results [3].

Our findings regarding risk factors for ESBL production are also similar to those reported in previous studies that included community-acquired UTIs and/or non-catheter-associated hospital-acquired UTIs. In our sample of hospitalized, catheterized patients, we found a strong positive association between prior treatment with beta-lactam and non-beta-lactam antibiotics and ESBL production, consistent with other studies of UTI caused by *E. coli* [2,6,8]. Like most other studies, we failed to detect significant differences in odds of ESBL by age, gender, comorbid conditions, and overall severity of illness; however we did find a significant positive association with urinary tract disease [6-8,10]. Patients who undergo a urinary tract procedure or surgery may be at greater risk for the emergence of resistance due in part to longer catheterization periods and increased antibiotic use. Nevertheless, urinary tract disease remained significant even after adjustment for length of catheterization and treatment with antibiotics, suggesting that it may have an independent association with ESBL.

While this is one of the largest studies to focus on CAUTI with ESBL-producing *E. coli* and document trends in resistance over time, the research does have some limitations. First, this study makes use of electronically available data to identify infections, as well as to determine patients’ clinical risk factors and comorbid health conditions. Information on preexisting health conditions was garnered from medical billing data, which are not collected for the purposes of research and may have low sensitivity and/or specificity. Aside from potential misclassification, using electronic data also prohibited us from reporting detailed information on the resistance patterns exhibited by the isolates. In addition, data on antimicrobial use was limited to those prescribed during the patients’ hospitalizations. The case-control study was restricted to three hospitals within a single, geographically narrow network, possibly limiting generalizability. Additionally, we were only able to investigate trends over time at one hospital due to limited data availability. Lastly, although our definition of CAUTI is consistent with NHSN guidelines of UTI onset >48 hours after catheterization, it is possible that some patients were bacteriuric prior to catheter insertion.

This study provides further evidence that treatment with beta-lactam and non-beta-lactam antibiotics is a risk factor for acquiring ESBL-producing *E. coli* CAUTI. Urinary tract disease is also identified as a risk factor, independent of antibiotic treatment and length of catheterization. Consistent with other reports, this study found an increase in ESBL prevalence among CAUTI in recent years. Risk factors for ESBL emergence may be different in CAUTI than in non-catheter-associated UTI.
